# Caffeine exposure during pregnancy, small for gestational age birth and neonatal outcome – results from the Norwegian Mother and Child Cohort Study

**DOI:** 10.1186/s12884-019-2215-9

**Published:** 2019-02-26

**Authors:** Dominika Modzelewska, Rino Bellocco, Anders Elfvin, Anne Lise Brantsæter, Helle Margrete Meltzer, Bo Jacobsson, Verena Sengpiel

**Affiliations:** 1 Institute of Clinical Sciences, Department of Obstetrics and Gynecology, University of Gothenburg, Sahlgrenska Academy, SE-416 85 Gothenburg, Sweden; 2 Department of Statistics and Quantitative Methods, University of Milano-Bicocca, Milan, Italy; 3 Department of Medical Epidemiology and Biostatistics, Karolinska Institutet, Stockholm, Sweden; 4 Department of Pediatrics, The Queen Silvia Children’s Hospital, Sahlgrenska University Hospital, Gothenburg, Sweden; 5 Department of Environmental Exposure and Epidemiology, Domain of Infection Control and Environmental Health, Norwegian Institute of Public Health, P.O. Box 4404 Nydalen, NO-0403 Oslo, Norway; 6 Department of Genetics and Bioinformatics, Domain of Health Data and Digitalisation, Institute of Public Health, Oslo, Norway; 7 Department of Obstetrics and Gynecology, Sahlgrenska University Hospital/Östra, SE-416 85 Gothenburg, Sweden

**Keywords:** Caffeine, Coffee, Neonatal outcome, Norwegian mother and child cohort study, Pregnancy, Small for gestational age

## Abstract

**Background:**

Maternal caffeine intake has repeatedly been linked to babies being born small for gestational age (SGA). SGA babies are known to be at increased risk for adverse neonatal outcomes. The aim of this study was to explore the associations between prenatal caffeine exposure and neonatal health.

**Methods:**

The study is based on 67,569 full-term singleton mother-infant pairs from the Norwegian Mother and Child Cohort Study. Caffeine consumption from different sources was self-reported in gestational week 22. Neonatal compound outcomes, namely (1) morbidity/mortality and (2) neonatal intervention, were created based on the Medical Birth Registry of Norway. Adjusted logistic regression was performed.

**Results:**

Caffeine exposure was associated to SGA (OR = 1.16, 95%CI: 1.10; 1.23) and being born SGA was significantly associated with neonatal health (OR = 3.09, 95%CI: 2.54; 3.78 for morbidity/mortality; OR = 3.94, 95%CI: 3.50; 4.45 for intervention). However, prenatal caffeine exposure was neither associated with neonatal morbidity/mortality (OR = 1.01, 95%CI: 0.96; 1.07) nor neonatal intervention (OR = 1.02, 95%CI: 1.00; 1.05 for a 100 mg caffeine intake increase). Results did not change after additional adjustment for SGA status.

**Conclusions:**

Moderate prenatal caffeine exposure (< 200 mg/day) does not seem to impair neonatal health, although prenatal caffeine exposure is associated with the child being born SGA and SGA with neonatal health. We suggest diversity in neonatal outcomes of SGA infants according to the underlying cause of low birth weight.

**Electronic supplementary material:**

The online version of this article (10.1186/s12884-019-2215-9) contains supplementary material, which is available to authorized users.

## Background

Caffeine is a plant alkaloid present in different types of beverages and food items such as coffee, tea, soft drinks and chocolate. Excessive caffeine consumption may cause health-related problems [1]. During pregnancy, maternal caffeine clearance is decreased [2], caffeine crosses the placenta easily, and the fetus relies mainly on maternal caffeine clearance. However, there are only few studies on the association of prenatal caffeine exposure and neonatal health. High caffeine intake during pregnancy may result in increased catecholamine levels in the fetus, which may cause placental vasoconstriction [3], and increased fetal heart rate, leading to impaired fetal oxygenation [4]. Some studies reported a higher risk of stillbirth, fetal death [5] or sudden infant death syndrome [6], while others reported no association between caffeine intake and infant death up to 1 year [7].

Our group published a significant association between prenatal caffeine exposure and reduced birth weight as well as increased risk of a baby being small for gestational age (SGA) (odds ratio (OR) = 1.18, 95% confidence interval (CI): 1.10, 1.26) based on 59,123 women from the Norwegian Mother and Child Cohort Study (MoBa) [8]. Consistent findings on the association between caffeine and SGA have been reported in several other studies [9–14].

The term SGA is used as a proxy for intrauterine growth restriction (IUGR). The etiology of IUGR often remains unclear. However, some common fetal, placental and maternal factors associated with IUGR and SGA have been proposed [15, 16]. SGA is strongly associated with neonatal morbidity and mortality [17], and SGA infants are more often admitted to Neonatal Intensive Care Units (NICUs) [18]. In obstetrics, SGA is often used as a surrogate outcome for IUGR and neonatal health as it is widely registered in neonatal records worldwide. However, depending on the underlying cause of reduced birth weight, the risk of neonatal morbidity and mortality might differ. Different underlying SGA causes have been suggested as an explanation for the so-called “birth weight paradox” – neonates born SGA by smokers have a lower infant mortality than neonates born SGA by non-smokers. Hernandez-Diaz proposed that neonates born SGA due to other - more severe SGA causes than smoking such as e.g. congenital malformation have a higher neonatal death risk [19].

Given the known association between caffeine and SGA as well as SGA and neonatal health, we hypothesize that maternal caffeine intake is associated to impaired neonatal health. However, the appropriateness of SGA as a surrogate outcome for IUGR and neonatal health has not yet been established for caffeine exposure studies.

The aim of this study is to assess the association between caffeine exposure from different sources, SGA birth and neonatal health in the Norwegian Mother and Child Cohort Study (MoBa), a large population based study with comprehensive information about lifestyle, maternal health and pregnancy-related conditions.

## Methods

### Study population and design

**Fig. 1:**
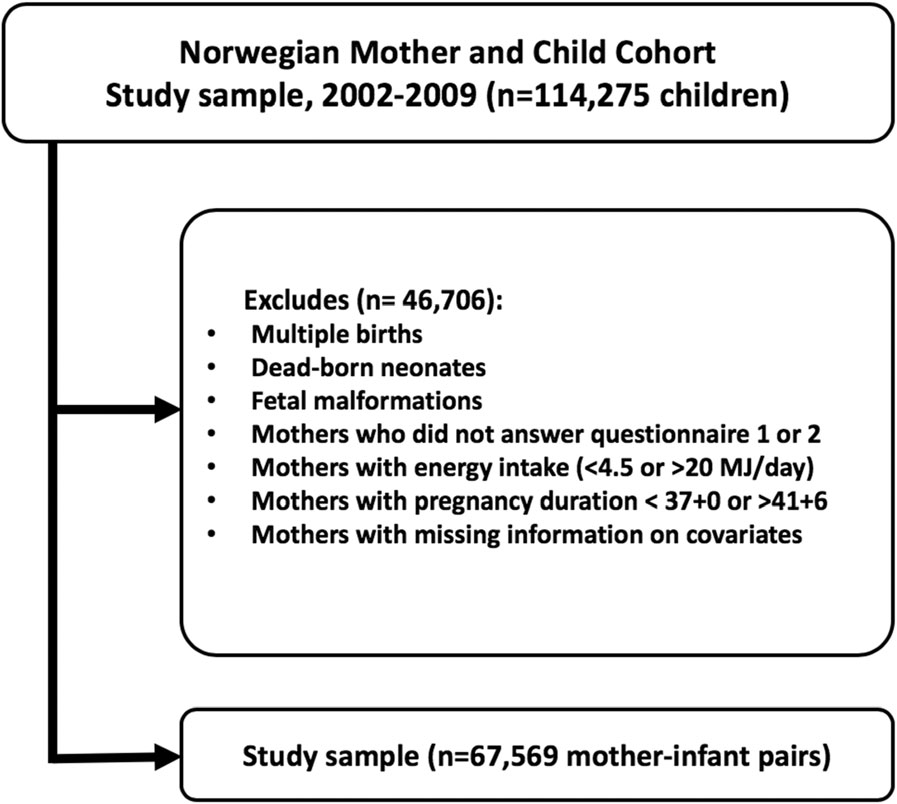
Study population flow chart. Flow chart of selection of pregnancies that met inclusion/exclusion criteria for the study population

This study is based on 67,569 mother-infant pairs from MoBa, an ongoing pregnancy cohort initiated and conducted by the Norwegian Institute of Public Health [20–22]. In short, MoBa is a prospective population-based study. Participants were recruited from all over Norway between 1999 and 2008. Of the invited women, 41% consented to participate. The cohort now includes 114,500 children, 95,200 mothers and 75,200 fathers. Follow-up is conducted by questionnaires at regular intervals and by linkage to national health registries such as the Medical Birth Registry of Norway (MBRN). Follow-up is conducted by questionnaires at regular intervals and by linkage to national health registries. The current study is based on version 8 of the quality-assured data files released for research in 2015 and uses information from the initial questionnaire about general health status and lifestyle filled out around gestational weeks 15 to 17, and the semi-quantitative food frequency questionnaire (FFQ) filled out around gestational week 22. Information from the Medical Birth Registry of Norway (MBRN) is integrated in the MoBa database. MBRN was established in 1967 and contains information about pregnancy, delivery, and health of the mother and the neonate for every live birth, stillbirth or induced abortion after the 12th week of gestation until discharge from the hospital [23]. Hospitals and birth institutions use a standard form to notify MBRN. The MBRN include information from the obstetric record, i.e. data filled in during antenatal visits to a general practitioner, midwife or obstetrician, and information from the medical record for inpatient care, i.e. data recorded from before the time of birth until discharge. The MBRN also contains data from neonatal and paediatric wards on congenital malformations, neonatal diagnoses and procedures performed on infants transferred to those units. The establishment and data collection in MoBa has obtained a license from the Norwegian Data Inspectorate and approval from The Regional Committee for Medical Research Ethics. The cohort is now based on regulations in the Norwegian Health Registry Act. Informed written consent was obtained from each participant. Out of the 114,275 children registered in MoBa, full-term lifeborn singletons without malformations have been selected, for further in- and exclusion criteria see Fig. 1. The current study was approved by the Regional Committee for Medical and Health Research Ethics South East (REK/Sør-Øst 2010/2683).

### Caffeine intake

Total daily caffeine intake as well as caffeine intake from various sources (e.g. coffee, tea, soft drinks and chocolate) was estimated based on the self-reported dietary habits in a validated food frequency questionnaire (FFQ) at gestational week 22. Beverage consumption in cups/glasses per day, week or month was reported in specified portion sizes: Coffee (filtered, instant, boiled/pressed, decaffeinated, caffé latte/cappuccino, espresso or fig/barley) 125 ml/cup, black tea 250 ml/cup, sugar-sweetened or diet cola, soft drinks or chocolate milk 250 ml/glass. Other caffeine sources reported were sandwich spread, desserts, cakes and sweets containing cocoa. Caffeine content was calculated based on FoodCalc [24] and the Norwegian Food Composition Table [25], for detailed information see Sengpiel et al. [8].

### Neonatal outcomes

In order to study neonatal outcomes, two composite variables were created: 1) neonatal morbidity or mortality and 2) neonatal intervention. Neonatal morbidity was defined as an Apgar score less than 4 after 5 min or the child being diagnosed with one of the following diagnoses registered in MBRN according to the International Classification of Disease, 10th Edition: birth asphyxia (P21), chronic respiratory disease originating in the perinatal period (P27), intracranial (nontraumatic) hemorrhage of fetus and newborn (P52), meconium ileus/necrotizing enterocolitis (P75, P76, P77, P78.0, P78.1), other disturbances of cerebral status of newborn (P91.0, P91.1, P91.2, P91.6) or retinopathy of prematurity (H35.1). Neonatal mortality was defined as death within 28 days after birth. The selection of the neonatal morbidities was based on the clinical knowledge regarding major conditions occurring among term, preterm and SGA infants. Malformations and genetic syndromes were not included. Neonatal intervention was defined based on data available from the MBRN as a newborn being transferred to the NICU, to receive respiratory or continuous positive airway pressure treatment or treatment with systemic antibiotics (Table 1).

**Table 1:** The prevalence (number and percentage) of different diagnosis within the neonatal compound outcome variables

	ICD 10 / disorder	N	%
Neonatal morbidity / mortality	total	2124	100
	APGAR score < 4	119	5.6
	Birth asphyxia (P21)	1962	92.4
	Other disturbances of cerebral status of newborn (P910–916)	21	1.0
	Intracranial (nontraumatic) haemorrhage of fetus and newborn	96	4.5
	Retinopathy of the prematurity (H351)	6	0.3
	Meconium ileus/necrotizing enterocolitis (P75, P76, P77, P780, P781)	13	0.6
	Chronic respiratory disease originating in the perinatal period (P27)	40	1.9
	Neonatal death	57	2.7
Neonatal intervention	total	9250	100
	Systemic antibiotics	1447	15.64
	Respirator	133	1.44
	CPAP	490	5.30
	NICU admission	9373	101.33

*ICD* International Classification of Disease, *CPAP* continuous positive airway pressure, *NICU* Neonatal intensive care unit. The same child can have different diagnosis or has become subject to several interventions so that the total number is lower than the sum of all diagnosis/interventions

### SGA

Information about birth weight and gestational age was obtained from the MBRN. SGA was defined according to Marsal as birthweight below 2 standard deviations (SD) of ultrasound-based growth curves [26]. Since there is no consensus on growth standards, we also ran supplemental analyses defining SGA according to the following approaches:

- Skjaerven, defining SGA as birthweight <10th percentile according to Norwegian population-based growth curves [27],

- Gardosi, defining SGA as birthweight <10th percentile according to customized ultrasound-based growth curves including information on maternal weight and height, parity and sex of the fetus [28].

### Covariates

Information on BMI, maternal smoking status, maternal alcohol consumption and occurrence of nausea, household income, preconceptional folic acid supplementation, and marital status was self-reported in the MoBa questionnaires. Information on maternal age at delivery and the baby’s sex was retrieved from the MBRN. The parity variable was based on combined data from MoBa and the MBRN, for detailed information see Sengpiel et al. [3]. Variables were categorized as presented in Table 2.

**Table 2:** Caffeine Intake According to Maternal Characteristics, n = 67,569, Norwegian Mother and Child Cohort Study, 2002–2009

			Total caffeine, mg/day		Coffee caffeine, mg/day		Tea caffeine, mg/day	
		N (%)	median (IQR)	p-value^a^	median (IQR)	p-value^a^	median (IQR)	p-value^a^
Household income, NOK	Both partners < 300,000	18,703 (28)	51 (20; 115)	< 0.05	3 (0; 57)	< 0.05	6 (0; 29)	< 0.05
	One partner ≥ 300,000	27,716 (42)	57 (23; 122)		6 (0; 66)		6 (1; 29)	
	Both partners ≥ 300,000	19,263 (29)	68 (29; 128)		13 (0; 85)		6 (1; 40)	
	Missing	1887	55 (21; 129)		3 (0; 61)		6 (0; 29)	
Maternal education, years	≤ 12	20,840 (32)	58 (22; 131)	< 0.05	3 (0; 66)	< 0.05	3 (0; 17)	< 0.05
	13–16	28,136 (43)	55 (22; 115)		7 (0; 63)		6 (0; 29)	
	17+	17,157 (26)	65 (28; 124)		13 (0; 85)		6 (3; 40)	
	Missing	1436	60 (22; 127)		7 (0; 69)		6 (0; 29)	
Marital status^b^	Yes	64,978 (96)	58 (23; 122)	< 0.05	8 (0; 68)	0.9	6 (1; 29)	< 0.05
	No	2591 (4)	60 (22; 142)		6 (0; 79)		3 (0; 17)	
Parity	0	35,631 (53)	48 (20; 102)	< 0.05	6 (0; 48)	< 0.05	6 (1; 29)	< 0.05
	1	20,664 (31)	68 (28; 137)		8 (0; 85)		6 (1; 40)	
	2	9080 (13)	85 (33; 175)		12 (0; 95)		6 (1; 40)	
	3+	2150 (3)	96 (38; 194)		20 (0;170)		6 (0; 40)	
	Missing	44	50 (19; 130)		9 (0; 92)		6 (1; 29)	
Maternal age, years	< 25	7712 (11)	35 (15; 82)	< 0.05	0 (0; 13)	< 0.05	3 (0; 17)	< 0.05
	25 to 29	22,969 (34)	48 (20; 102)		5 (0; 44)		6 (1; 29)	
	30 to 34	28,699 (42)	69 (28; 135)		12 (0; 85)		6 (1; 40)	
	> 34	8189 (12)	92 (40; 180)		28 (0; 129)		6 (1; 40)	
Alcohol consumption, units/week	No alcohol	60,155 (89)	54 (22; 115)	< 0.05	6 (0; 61)	< 0.05	6 (1; 29)	< 0.05
	< 0.5	6214 (9)	94 (45; 176)		38 (5; 119)		17 (3; 40)	
	> 0.5	1199 (2)	128 (66; 218)		76 (13; 173)		17 (3; 40)	
Smoking status	Daily	3596 (5)	157 (59; 258)	< 0.05	78 (0; 179)	< 0.05	3 (0; 17)	< 0.05
	Occasionally	1816 (3)	110 (45; 201)		44 (0; 170)		3 (0; 17)	
	Never	61,778 (92)	55 (22; 113)		6 (0; 61)		6 (1; 29)	
	Missing	379	65 (28; 130)		12 (0; 75)		6 (0; 40)	
Nausea	No	59,877 (89)	61 (24; 126)	< 0.05	8 (0; 79)	< 0.05	6 (1; 29)	< 0.05
	Yes	7692 (11)	45 (16; 99)		0.1 (0; 21)		6 (0; 29)	
Folic acid supplementation	No	37,454 (55)	62 (24; 131)	< 0.05	7 (0; 82)	0.86	6 (1; 29)	< 0.05
	Yes	30,115 (45)	55 (22; 112)		8 (0; 63)		6 (1; 29)	
Planned pregnancy	No	12,994 (19)	61 (23; 133)	< 0.05	7 (0; 83)	0.57	6 (0; 29)	< 0.05
	Yes	54,575 (81)	58 (23; 120)		8 (0; 67)		6 (1; 29)	
Baby’s sex	Girl	34,340 (51)	59 (23; 124)	0.13	7 (0; 69)	0.98	6 (1; 29)	0.88
	Boy	33,229 (49)	58 (23; 122)		8 (0; 68)		6 (1; 29)	
Quartiles of total energy intake (kcal)	< 1875	16,882 (25)	42 (15; 95)	< 0.05	4 (0; 45)	< 0.05	3 (0; 20)	< 0.05
	1875–2222	16,883 (25)	53 (21; 112)		7 (0; 64)		5 (1; 28)	
	2223–2651	16,927 (25)	60 (25; 125)		8 (0; 72)		6 (1; 31)	
	> 2651	16,877 (25)	78 (32; 158)		10 (0; 89)		6 (1; 34)	
BMI	< 18.5	2046 (3)	56 (22; 114)	< 0.05	7 (0; 64)	< 0.05	6 (1; 28)	< 0.05
	18.5 to 24.9	43,713 (65)	59 (23; 122)		8 (0; 74)		6 (1; 28)	
	25 to 29.9	15,206 (22)	59 (23; 126)		6 (0; 66)		6 (1; 28)	
	30 to 34.9	4647 (7)	56 (21; 124)		3 (0; 48)		3 (0; 17)	
	35 to 39.9	1358 (2)	51 (20; 114)		2 (0; 36)		3 (0; 17)	
	> 40	406 (1)	60 (21; 128)		0 (0, 27)		3 (0; 17)	
	Missing	193	70 (25; 137)		6 (0; 62)		6 (0; 40)	

*IQR* interquartile range, ^a^Kruskal-Wallis test, ^b^Marital Status is defined as either married/cohabiting or not

### Statistical analysis

Caffeine intake according to maternal characteristics was analyzed by the Kruskal-Wallis test.

The overall association between caffeine intake and neonatal outcomes was analyzed using a logistic regression model, both crude and adjusted for the following categorized variables: maternal pre-pregnancy BMI, household income, maternal education, marital status, parity, maternal age at delivery, smoking status, presence of nausea, folic acid supplementation, planned pregnancy, baby’s sex and total energy intake. Missing data were treated as a category of its own. Additionally, logistic regression analyses were performed including SGA into the model to capture possible indirect effects of prenatal caffeine exposure. All coefficients were reported for a 100 mg change in daily caffeine intake, which equals approximately one cup of coffee. The correlation between covariates was analysed to consider possible collinearity.

Additionally, the assumption of a linear relationship between maternal caffeine intake and log odds of the outcome was assessed by flexible models based on restricted cubic spline regression with five fixed knots.

All statistical analyses were performed using SPSS version 22 and R version 3.3.1.

## Results

### Caffeine intake

**Fig. 2:**
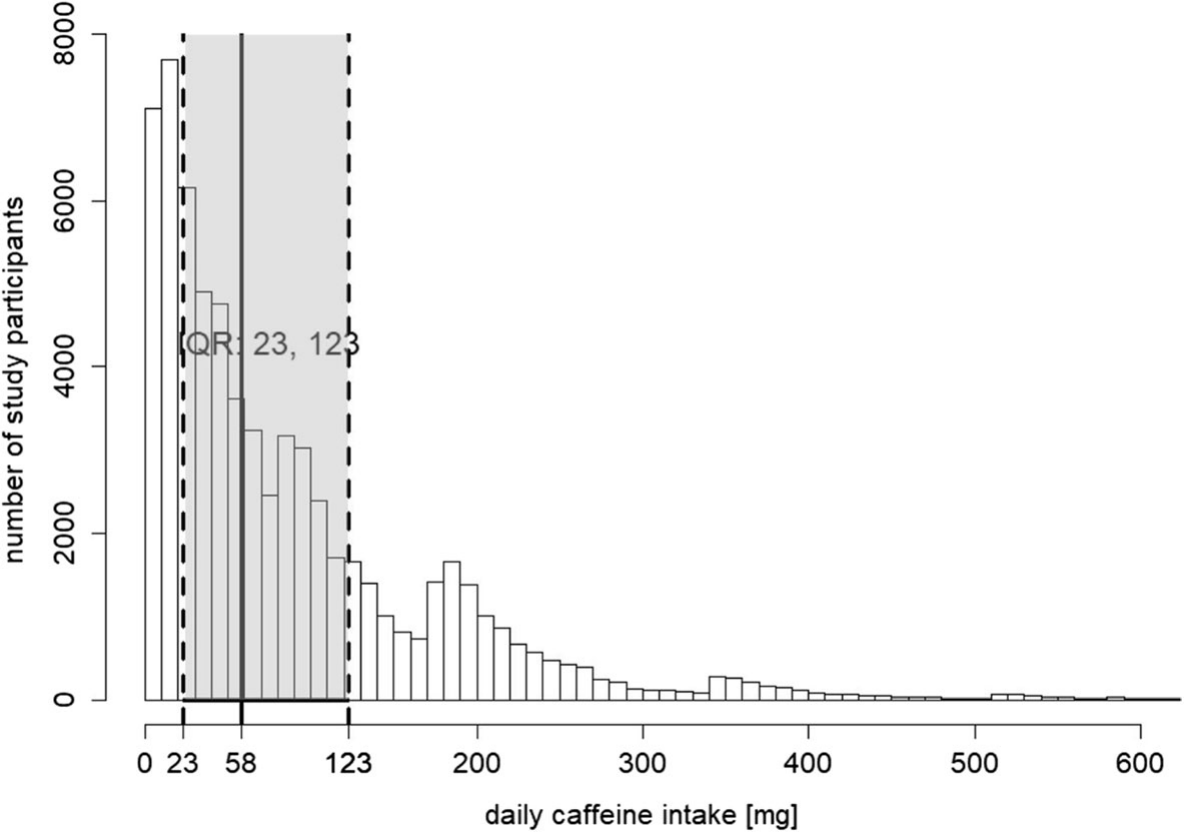
Distribution of total daily caffeine intake. Describes the distribution of daily caffeine consumption, median and interquartile range marked in grey, *n* = 67,569, in the Norwegian Mother and Child Cohort Study 2002 to 2009

Daily caffeine intake varied between 0 and 1843 mg, with a median value at 58 mg (approximately half a cup of coffee), and 75% of the women consumed less than 123 mg of caffeine per day Fig. 2. Caffeine intake according to maternal characteristics is presented in Table 2. Women who were older, more educated, smoked and had higher incomes consumed more caffeine than those who were younger, non-smoking and had lower incomes.

**Fig. 3:**
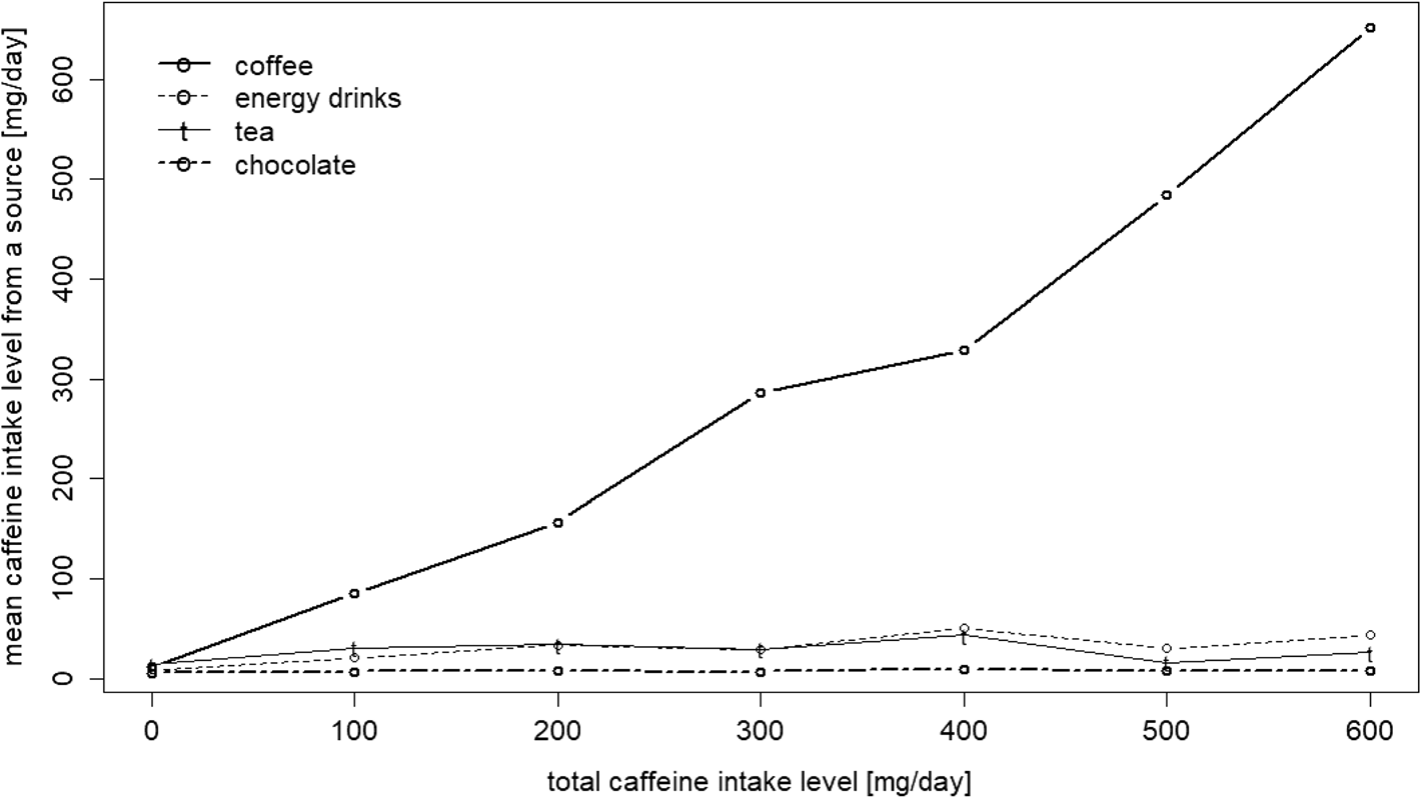
Caffeine intake from different sources according to total caffeine intake. Shows the mean caffeine intake from different sources (coffee, tea, soft drinks and chocolate) over the whole distribution of total caffeine intake (in mg/day)

The main source of caffeine varied across total caffeine intake. Among low caffeine consumers (< 100 mg/d), a large portion of caffeine originated from tea, caffeinated soft drinks and chocolate (Fig. 3). With increasing total caffeine intake the fraction of caffeine from coffee increased considerably (Pearson correlation = 0.56, *P* < 0.05).

### Caffeine exposure and SGA

The median birth weight in the study population was 3600 g (interquartile range: 3270 g; 3935 g) and 2% of all babies were born SGA. Consistent with our previous study [8], caffeine exposure was associated to SGA (OR = 1.16, 95%CI: 1.10; 1.23). Comparable associations were found if SGA was defined according to Gardosi (14%, OR = 1.1, 95%CI: 1.07; 1.13) or Skjaerven (8%, OR = 1.13, 95%CI: 1.1; 1.17).

### SGA and neonatal outcome

The prevalence of the neonatal outcomes is presented in Table 3. As expected, infants born SGA had increased odds for both neonatal morbidity/mortality and neonatal intervention (OR = 3.09, 95%CI: 2.54; 3.78 and OR = 3.94, 95%CI: 3.50; 4.45, respectively, Similar results were found for the other SGA definitions, see Additional file 1).

**Table 3:** Prevalence of Neonatal Outcomes According to Quartiles of Total Caffeine Intake in the Norwegian Mother and Child Cohort Study, 2002–2009

			Low caffeine intake (< 23 mg/day)	Medium caffeine intake (23–58 mg/day)	High caffeine intake (59–123 mg/day)	Very high caffeine intake (> 123 mg/day)
	n total		16,893	16,892	16,892	16,892
Neonatal morbidity/mortality	no	65,445	16,356	16,349	16,362	16,378
	n (%)	(97)	(97)	(97)	(97)	(97)
	yes	2124	537	543	530	514
	n (%)	(3)	(3)	(3)	(3)	(3)
Neonatal intervention	no	58,319	14,559	14,591	14,570	14,599
	n (%)	(86)	(86)	(86)	(86)	(86)
	yes	9250	2334	2301	2322	2293
	n (%)	(14)	(14)	(14)	(14)	(14)

### Prenatal caffeine exposure and neonatal outcomes

Total caffeine intake was not significantly associated with neonatal morbidity/mortality (OR = 1.01, 95%CI: 0.96; 1.07), or neonatal intervention (OR = 1.02, 95% CI: 1.00; 1.05). Additional adjustment for SGA did not affect the estimates (OR = 1.01, 95% CI: 0.95;1.06 for neonatal morbidity/mortality; OR = 1.02, 95% CI: 0.99; 1.05 for neonatal intervention) with similar results for the other SGA definitions, see Additional file 2. Restricting the analysis to never-smokers (*n* = 61,778) did not change the results (neonatal morbidity/mortality; OR = 1.00, 95% CI: 0.93; 1.06; neonatal intervention OR = 1.02, 95% CI: 0.99; 1.06). Neither did adding interaction terms for maternal BMI, age or the child’s sex change the association (results not shown). No significant association between the different caffeine sources and neonatal outcome variables was found, except for caffeine from chocolate. In the adjusted model, 100 mg increase in chocolate caffeine intake was associated with increased odds for neonatal intervention (OR = 1.59, 95%CI: 1.07; 2.36), see Table 4. There was no evidence of a nonlinear relationship between total caffeine intake (or caffeine intake from different sources) and log odds of neonatal outcomes (*P* > 0.05).

**Table 4:** Association Between Caffeine Intake and Neonatal Outcomes, n = 67,569, Norwegian Mother and Child Cohort Study, 2002–2009

	Total caffeine intake			Coffee caffeine intake			Tea caffeine intake			Soft drink caffeine intake			Chocolate caffeine intake		
	OR		*p*-value	OR		*p*-value	OR		*p*-value	OR		*p*-value	OR		*p*-value
	95% CI			95% CI			95% CI			95% CI			95% CI		
Unadjusted:															
Neonatal morbidity/mortality	0.93		0.01	0.93		0.01	0.89		0.14	1.03		0.73	0.37		0.01
	0.89	0.98		0.88	0.98		0.77	1.04		0.88	1.19		0.17	0.79	
Neonatal intervention	0.98		0.05	0.97		0.06	0.93		0.07	1.04		0.30	0.83		0.32
	0.95	1.00		0.95	1.00		0.87	1.01		0.96	1.12		0.58	1.19	
Adjusted:															
Neonatal morbidity/mortality	1.00		0.88	1.00		0.83	1.04		0.66	1.05		0.54	0.92		0.84
	0.95	1.06		0.94	1.06		0.88	1.21		0.88	1.23		0.41	1.99	
Neonatal intervention	1.02		0.07	1.02		0.25	0.99		0.85	1.08		0.05	1.51		0.03
	1.00	1.05		0.99	1.05		0.91	1.07		1.00	1.17		1.03	2.19	

*CI* confidence interval, *OR* odds ratios, Odds ratios for the outcomes of interest as a function of a 100-mg change in daily caffeine intake. ORs according to logistic regression, both unadjusted and adjusted for maternal pre-pregnancy body mass index, household income, maternal education, marital status, parity, maternal age at delivery, smoking status, presence of nausea, folic acid supplementation, planned pregnancy, baby’s sex and total energy intake. When studying different caffeine sources, analyses were mutually adjusted for caffeine sources

## Discussion

The main finding of this study is the lack of any statistically significant association between moderate maternal caffeine intake and neonatal health studied in form of compound neonatal morbidity/mortality and neonatal intervention variables. We previously found a statistically significant association between prenatal caffeine exposure and SGA [8]. Further, we found that SGA was significantly associated with both neonatal outcome variables. While we expected to find a significant association between total caffeine intake and neonatal health, either due to fully (Fig. 4.1) or partly SGA-mediated effects (Fig. 4.2) or caused by residual confounding (Fig. 4.3 or 4.4), maternal caffeine consumption was not associated with neonatal health. Neither did additional adjustment for SGA impact on the association between caffeine exposure and neonatal health. An alternative explanation for our findings would be that the effect of SGA on neonatal intervention differs depending on the underlying cause of SGA (Fig. 4.5). Obviously, spurious findings as shown in Fig. 4.3 and 4.4 could be integrated in Fig. 4.5 as well. Assumed there are different types of SGA, one type being statistically – not necessarily causal – linked to maternal caffeine intake and another type due to other – more serious causes for SGA, the neonates being born SGA in association with prenatal caffeine exposure seem to perform better than neonates being born SGA due to other causes. A similar conclusion was presented by Hernandez-Diaz proposing that neonates born SGA due to maternal smoking might have a lower risk of neonatal death than neonates born SGA due to other more severe causes such as congenital malformation [19]. These findings warrant caution when using SGA as a proxy for impaired neonatal health in epidemiologic studies. However, when interpreting the results, it is important to keep in mind that 75% of the study population had caffeine consumption below 123 mg/day, and only 3% of the women had a caffeine intake above 300 mg/day. Therefore, no conclusions can be drawn regarding the effect of high caffeine intake on neonatal health. Aversion to coffee in addition to nausea is a common symptom in early pregnancy resulting in substantially lower caffeine intake in early pregnancy than before pregnancy, and this was also found in our cohort [8].

**Fig. 4:**
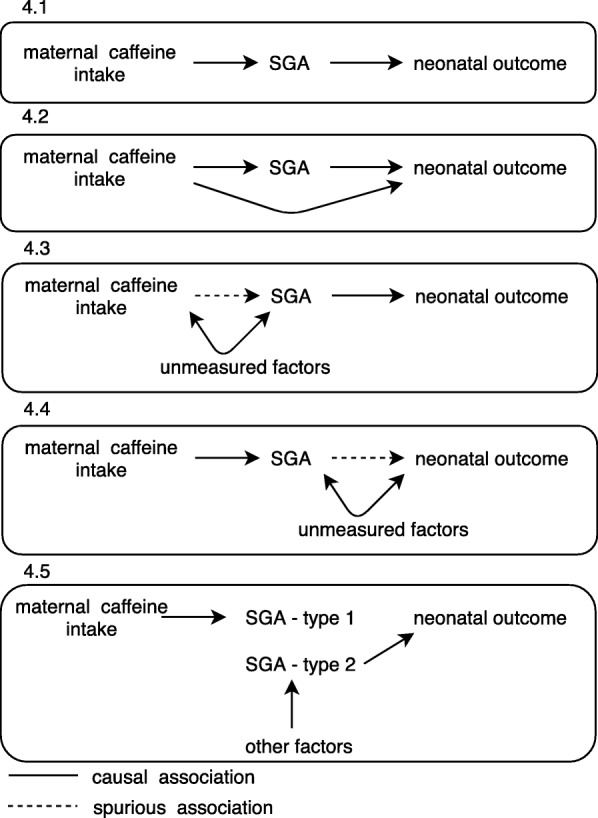
Possible causal structures for the association of maternal caffeine intake, birth weight/SGA, and neonatal intervention. The figure presents four different directed acyclic graphs illustrating possible associations between maternal caffeine intake, birth weight/SGA and neonatal intervention

Results for the different caffeine sources gave the same results except for caffeine from chocolate, which was associated with an increased risk for neonatal intervention in the adjusted analyses. We have no explanation for this and the finding for chocolate is probably due to chance or residual confounding. Habitual intake of coffee and tea is easier to recall than intakes of caffeine-containing soft drinks, chocolate containing foods and plain chocolate as consumption of these items is less regular than coffee and tea. Furthermore, individuals tend to underreport foods perceived as unhealthy more than foods perceived as healthy. Estimated intake of caffeine from chocolate was low, 50% of the women had chocolate caffeine intake lower than 4.3 mg/day (interquartile range: 2.2–9.5 mg/day). Thus, the chocolate caffeine variable is less comparable to the other variables and might capture other qualities of a woman’s dietary habits such as having snacks between meals, amount of sweets consumed etc.

To our knowledge, only few studies have evaluated the effect of prenatal caffeine exposure on neonatal health other than birth weight – e.g. neonatal death, sudden infant death and neonatal heart rate. Some studies focused on stillbirth. However, we did not find any studies with comparable outcomes as in this study. A small study from Finland (*n* = 20) suggests that high caffeine intake during pregnancy (300–600 mg/day, equal to 3–6 cups of coffee/day) may increase catecholamine levels in the fetus and lead to placental vasoconstriction [3]. A study by Resh et al. presented increased contraction rates of surgically isolated fetal hearts when kept in a nutrient solution containing up to 1 mmol/L caffeine. The authors suggested a possible effect of moderate to excessive intake of caffeine during pregnancy on irregular fetal heart rate, leading to impaired fetal oxygenation [4].

A metanalysis by Chen et al. found an increased risk for pregnancy loss (miscarriage or stillbirth) for maternal caffeine intake above 350 mg/day but not for lower levels [5]. Based on 88,482 women of the Danish National Birth Cohort, Bech et al. published an association between high coffee intake during pregnancy (more than 8 cups/day) and fetal death. However, tea and cola consumption was not associated with fetal death arguing against caffeine being the causal link [29]. In a case-control study from New Zealand (*n* = 393 cases and *n* = 1592 controls), caffeine intake from coffee, tea and cola above 400 mg/day was associated with increased risk for sudden infant death. However, the caffeine intake was assessed retrospectively, opening for recall bias [6]. A prospective Danish cohort study (*n* = 18,478), found no association between caffeine intake and infant death during the first year of life [7].

### Strength and limitations

To our knowledge, with a sample size of 67,569, this study is the largest study performed on the association between caffeine intake and neonatal health. Although the MoBa participation rate is 41%, and the MoBa population differs from the general population of pregnant women by some exposure and outcome characteristics, Nilsen et al. found no differences in eight selected exposure-outcome associations between MoBa participants and the general population registered in the MBRN [22].

The comprehensive MoBa dataset enabled us to control for many important covariates. When studying the potential effects of caffeine exposure, it is crucial to adjust for smoking. Although smoking status was self-reported, the variable has been evaluated against the biological marker cotinine and shown acceptable validity [30]. However, residual confounding cannot be ruled out.

A strength of the MoBa FFQ is that different caffeine sources, portion sizes and preparation methods for coffee could be considered for the estimation of daily caffeine intake. We found similar results for the different caffeine sources studied strengthening the overall finding for prenatal caffeine exposure. Self-reported food consumption might lead to incorrect nutrient intake estimation. However, the MoBa FFQ has been extensively validated based in a sub-population (*n* = 119), using four-day weighed food diaries and several biomarkers as reference measures. The agreement between the FFQ and the food diaries was particularly high for coffee and tea intake (r = 0.53, r = 0.80 respectively), which are the main sources of caffeine in our study population [31, 32]. Furthermore, the prospective data collection for caffeine intake ensured that a woman’s response was not influenced by her knowledge of the pregnancy outcome.

Neonatal diagnoses were obtained from MBRN, ensuring that diagnoses were registered as given by healthcare professionals. As some of the neonatal conditions might not be diagnosed before the baby leaves the hospital, some of these diagnoses may have been missed. However, the neonatal intervention variable, indicating that the neonate needed medical help in some way, gives complete information for the time until discharge from the hospital.

Supplemental analyses for different common SGA definitions facilitate the interpretation and adaptation of the results in different research settings and healthcare systems.

The use of a continuous scale for caffeine intake, as opposed to categorization, ensures that there is no loss of information, power reduction or dilution of the effect of caffeine on neonatal outcome. Categorization risks exposure to misclassification and hampers the comparability among studies. To investigate threshold effects of the exposure, we analysed the possible dose–effect relationship of caffeine intake by restricted cubic spline regression rather than in different categories of caffeine intake.

## Conclusion

Moderate maternal caffeine intake is associated with the baby being born SGA, and SGA is strongly associated with impaired neonatal health. However, no significant associations between maternal caffeine intake and neonatal health were found. We suggest that different SGA entities are associated with caffeine intake and neonatal health respectively. Prenatal caffeine exposure seems to be associated with a less severe type of SGA that is not consistently associated to adverse neonatal outcome. These findings warrant caution when using SGA as a proxy for impaired neonatal health in epidemiologic studies. However, when interpreting the results, the relatively low caffeine intake in the cohort, with 75% of the participants consuming less than 123 mg/day, must be kept in mind.

## Additional files

Additional file 1: Association Between Small for Gestational Age (SGA) and Neonatal Outcomes, *n* = 67,569, Norwegian Mother and Child Cohort Study, 2002–2009. aOR – adjusted odds ratios, CI – confidence interval, SGA - small for gestational age. The table shows adjusted odds ratios for the association of small for gestational age (according to different definitions of SGA: Gardosi, Skjaerven and Marsal) with neonatal outcomes. The Wald 95% confidence intervals are provided for each estimate. ORs are adjusted for: maternal pre-pregnancy body mass index, household income, maternal education, marital status, parity, maternal age at delivery, smoking status, presence of nausea, folic acid supplementation, planned pregnancy and baby’s sex. (DOCX 22 kb)

Additional file 2: Association between caffeine intake and neonatal outcomes, additional adjustment for SGA. CI – confidence interval, OR – odds ratio, SGA – small for gestational age. OR for the outcomes of interest as a function of 100 mg change in total caffeine intake. ORs are adjusted for: maternal pre-pregnancy body mass index, household income, maternal education, marital status, parity, maternal age at delivery, smoking status, presence of nausea, folic acid supplementation, planned pregnancy, baby’s sex, total energy intake, with additional adjustment for small for gestational age (according to a given definition of small for gestational age). n = 67,569, in the Norwegian Mother and Child Cohort Study 2002 to 2009. (DOCX 19 kb)

## Abbreviations

BMI: Body mass index
CI: Confidence interval
FFQ: Food frequency questionnaire
IUGR: Intrauterine growth restriction
MBRN: Medical Birth Register of Norway
MoBa: Norwegian Mother and Child Cohort Study
NICU: Neonatal Intensive Care Units
OR: Odds ratio
SGA: Small for gestational age
