# Supplementary material for: Caffeine exposure during pregnancy, small for gestational age birth and neonatal outcome – results from the Norwegian Mother and Child Cohort Study
Source: BMC Pregnancy Childbirth. 2019 Feb 26;19:80. doi: 10.1186/s12884-019-2215-9 (PMC6390347; doi:10.1186/s12884-019-2215-9)
Supplement: Supplementary file 1 — Association Between Small for Gestational Age (SGA) and Neonatal Outcomes, n = 67,569, Norwegian Mother and Child Cohort Study, 2002–2009. aOR – adjusted odds ratios, CI – confidence interval, SGA - small for gestational age. The table shows adjusted odds ratios for the association of small for gestational age (according to different definitions of SGA: Gardosi, Skjaerven and Marsal) with neonatal outcomes. The Wald 95% confidence intervals are provided for each estimate. ORs are adjusted for: maternal pre-pregnancy body mass index, household income, maternal education, marital status, parity, maternal age at delivery, smoking status, presence of nausea, folic acid supplementation, planned pregnancy and baby’s sex. (DOCX 22 kb) [file 12884_2019_2215_MOESM1_ESM.docx]

### Additional file 1 Association Between Small for Gestational Age (SGA) and Neonatal Outcomes, n=67,569, Norwegian Mother and Child Cohort Study, 2002-2009.

|  | Neonatal morbidity/mortality | | | | | Neonatal intervention | | | | | |
| --- | --- | --- | --- | --- | --- | --- | --- | --- | --- | --- | --- |
|  | no. of SGA cases among cases  (absolute risk) | | aOR | 95% CI | | no. of SGA cases among cases  (absolute risk) | | aOR | 95% CI | | |
| SGA Gardosi | no | yes | 1.37 | 1.22 | 1.53 | no | yes | 1.41 | 1.33 | 1.50 | |
|  | 1658 | 413 |  |  |  | 7399 | 1730 |  |  |  |  |
|  | (20%) | |  |  |  | (19%) | |  |  | | |
|  | (20%) | |  |  |  |  |  |  |  |  |  |
| SGA Skjaerven | no | yes | 1.51 | 1.32 | 1.68 | no | yes | 1.56 | 1.45 | | 1.68 |
|  | 1834 | 285 |  |  |  | 8073 | 1169 |  |  |  |  |
|  | (16%) | |  |  |  | (13%) | |  |  | | |
| SGA Marsal | no | yes | 3.07 | 2.50 | 3.74 | no | yes | 3.92 | 3.47 | 4.42 | |
|  | 1993 | 126 |  |  |  | 8740 | 502 |  |  |  |  |
|  | (6%) | |  |  | | (5%) | |  |  | | |

aOR – adjusted odds ratios, CI – confidence interval, SGA - small for gestational age. The table shows adjusted odds ratios for the association of small for gestational age (according to different definitions of SGA: Gardosi, Skjaerven and Marsal) with neonatal outcomes. The Wald 95% confidence intervals are provided for each estimate. ORs are adjusted for: maternal pre-pregnancy body mass index, household income, maternal education, marital status, parity, maternal age at delivery, smoking status, presence of nausea, folic acid supplementation, planned pregnancy and baby’s sex.
