# Supplementary material for: Caffeine exposure during pregnancy, small for gestational age birth and neonatal outcome – results from the Norwegian Mother and Child Cohort Study
Source: BMC Pregnancy Childbirth. 2019 Feb 26;19:80. doi: 10.1186/s12884-019-2215-9 (PMC6390347; doi:10.1186/s12884-019-2215-9)
Supplement: Supplementary file 2 — Association between caffeine intake and neonatal outcomes, additional adjustment for SGA. CI – confidence interval, OR – odds ratio, SGA – small for gestational age. OR for the outcomes of interest as a function of 100 mg change in total caffeine intake. ORs are adjusted for: maternal pre-pregnancy body mass index, household income, maternal education, marital status, parity, maternal age at delivery, smoking status, presence of nausea, folic acid supplementation, planned pregnancy, baby’s sex, total energy intake, with additional adjustment for small for gestational age (according to a given definition of small for gestational age). n = 67,569, in the Norwegian Mother and Child Cohort Study 2002 to 2009. (DOCX 19 kb) [file 12884_2019_2215_MOESM2_ESM.docx]

### Additional file 2 Association between caffeine intake and neonatal outcomes, additional adjustment for SGA.

|  | Total caffeine intake | | | Coffee caffeine intake | | | Tea caffeine intake | | | Soft drink caffeine intake | | | Chocolate caffeine intake | | |
| --- | --- | --- | --- | --- | --- | --- | --- | --- | --- | --- | --- | --- | --- | --- | --- |
|  | aOR | 95% CI | p-value | aOR | 95% CI | p-value | aOR | 95% CI | p-value | aOR | 95% CI | p-value | aOR | 95% CI | p-value |
| SGA Marsal |  |  |  |  |  |  |  |  |  |  |  |  |  |  |  |
| Neonatal morbidity / mortality | 0.99 | 0.94; 1.05 | 0.84 | 1.00 | 0.93; 1.05 | 0.76 | 1.00 | 0.86; 1.18 | 0.93 | 1.05 | 0.89; 1.24 | 0.53 | 0.93 | 0.42; 2.02 | 0.87 |
| Neonatal intervention | 1.02 | 0.99; 1.04 | 0.22 | 1.01 | 0.98; 1.05 | 0.36 | 0.96 | 0.89; 1.04 | 0.36 | 1.07 | 0.98; 1.16 | 0.11 | 1.54 | 1.05; 2.25 | 0.02 |
| SGA SKjaerven |  |  |  |  |  |  |  |  |  |  |  |  |  |  |  |
| Neonatal morbidity / mortality | 1.00 | 0.95; 1.06 | 0.98 | 0.99 | 0.93; 1.05 | 0.77 | 1.04 | 0.87; 1.19 | 0.82 | 1.06 | 0.89; 1.24 | 0.50 | 0.91 | 0.41; 1.96 | 0.81 |
| Neonatal intervention | 1.02 | 0.99; 1.05 | 0.19 | 1.01 | 0.98; 1.05 | 0.36 | 0.97 | 0.90; 1.05 | 0.50 | 1.07 | 0.99; 1.16 | 0.09 | 1.51 | 1.03; 2.19 | 0.03 |
| SGA Gardosi |  |  |  |  |  |  |  |  |  |  |  |  |  |  |  |
| Neonatal morbidity / mortality | 1.00 | 0.94; 1.06 | 0.96 | 0.99 | 0.92; 1.05 | 0.65 | 1.02 | 0.87; 1.20 | 0.78 | 1.07 | 0.90; 1.26 | 0.41 | 0.98 | 0.44; 2.13 | 0.98 |
| Neonatal intervention | 1.02 | 0.99; 1.05 | 0.12 | 1.01 | 0.99; 1.05 | 0.26 | 0.97 | 0.90; 1.05 | 0.50 | 1.08 | 0.99; 1.17 | 0.06 | 1.53 | 1.05; 2.23 | 0.03 |

CI – confidence interval, OR – odds ratio, SGA – small for gestational age. OR for the outcomes of interest as a function of 100 mg change in total caffeine intake. ORs are adjusted for: maternal pre-pregnancy body mass index, household income, maternal education, marital status, parity, maternal age at delivery, smoking status, presence of nausea, folic acid supplementation, planned pregnancy, baby’s sex, total energy intake, with additional adjustment for small for gestational age (according to a given definition of small for gestational age). n=67,569, in the Norwegian Mother and Child Cohort Study 2002 to 2009.
